# The Precursor to Glutathione (GSH), γ-Glutamylcysteine (GGC), Can Ameliorate Oxidative Damage and Neuroinflammation Induced by Aβ_40_ Oligomers in Human Astrocytes

**DOI:** 10.3389/fnagi.2019.00177

**Published:** 2019-08-08

**Authors:** Nady Braidy, Martin Zarka, Bat-Erdene Jugder, Jeffrey Welch, Tharusha Jayasena, Daniel K. Y. Chan, Perminder Sachdev, Wallace Bridge

**Affiliations:** ^1^Centre for Healthy Ageing, School of Psychiatry, Faculty of Medicine, University of New South Wales, Sydney, NSW, Australia; ^2^School of Biotechnology and Biomolecular Sciences, Faculty of Science, University of New South Wales, Sydney, NSW, Australia; ^3^Faculty of Medicine, University of New South Wales, Sydney, NSW, Australia; ^4^Department of Aged Care and Rehabilitation, Bankstown Hospital, Bankstown, NSW, Australia; ^5^Neuropsychiatric Institute, Euroa Centre, Prince of Wales Hospital, Sydney, NSW, Australia

**Keywords:** Alzheimer’s disease, glutathione, oxidative stress, antioxidants, dementia

## Abstract

Glutathione (GSH) is one of the most abundant thiol antioxidants in cells. Many chronic and age-related diseases are associated with a decline in cellular GSH levels or impairment in the catalytic activity of the GSH biosynthetic enzyme glutamate cysteine ligase (GCL). γ-glutamylcysteine (GGC), a precursor to glutathione (GSH), can replenish depleted GSH levels under oxidative stress conditions, by circumventing the regulation of GSH biosynthesis and providing the limiting substrate. Soluble amyloid-β (Aβ) oligomers have been shown to induce oxidative stress, synaptic dysfunction and memory deficits which have been reported in Alzheimer’s disease (AD). Calcium ions, which are increased with age and in AD, have been previously reported to enhance the formation of Aβ_40_ oligomers, which have been casually associated with the pathogenesis of the underlying neurodegenerative condition. In this study, we examined the potential beneficial effects of GGC against exogenous Aβ_40_ oligomers on biomarkers of apoptosis and cell death, oxidative stress, and neuroinflammation, in human astrocytes. Treatment with Aβ_40_ oligomers significantly reduced the cell viability and apoptosis of astrocyte brain cultures and increased oxidative modifications of DNA, lipids, and protein, enhanced pro-inflammatory cytokine release and increased the activity of the proteolytic matrix metalloproteinase enzyme, matric metalloproteinase (MMP)-2 and reduced the activity of MMP-9 after 24 h. Co-treatment of Aβ_40_ oligomers with GGC at 200 μM increased the activity of the antioxidant enzymes superoxide dismutase (SOD) and glutathione peroxidase (GPx) and led to significant increases in the levels of the total antioxidant capacity (TAC) and GSH and reduced the GSSG/GSH ratio. GGC also upregulated the level of the anti-inflammatory cytokine IL-10 and reduced the levels of the pro-inflammatory cytokines (TNF-α, IL-6, and IL-1β) and attenuated the changes in metalloproteinase activity in oligomeric Aβ_40_-treated astrocytes. Our data provides renewed insight on the beneficial effects of increased GSH levels by GGC in human astrocytes, and identifies yet another potential therapeutic strategy to attenuate the cytotoxic effects of Aβ oligomers in AD.

## Introduction

Alzheimer’s disease (AD) is the most common form of dementia affecting the elderly. Extracellular deposition of β-amyloid (Aβ plaques), intraneuronal tau accumulation, inflammation (activated astrocytes and microglia), and neuronal loss are all consistent pathological features of the disease (Porquet et al., 2015). Unlike Aβ plaques, inflammation correlates with neuronal loss and cognitive decline in AD, suggesting it plays an important role in disease progression (Wang et al., 2015; Wilkins et al., 2015).

It is well established that Aβ peptides existed in both fibrillary and non-fibrillar forms (Poduslo and Howell, 2015; Zhang et al., 2015). Soluble oligomeric Aβ aggregates have been shown to bind specifically to synapses in differentiated hippocampal neuronal cultures. These oligomers are capable of disrupting long-term potentiation, a classic experimental paradigm for memory and synaptic plasticity (Izzo et al., 2014; Nguyen and Derreumaux, 2014). Small soluble Aβ oligomers are now considered the primary neurotoxic entity in AD. Several studies have shown that both oligomeric Aβ_42_ and Aβ_40_ are both neurotoxic using both human and murine neuronal cell cultures. Moreover, there is a strong association between the level of soluble oligomeric Aβ, and the severity of synaptic loss and cognitive dysfunction in AD when compared to their fibrillar counterpart (Lesne, 2014; Nguyen et al., 2014; Wang et al., 2015). Similarly, a number of early-onset familial AD mutations display a more aggressive disease course and a greater propensity for Aβ to form soluble oligomeric Aβ aggregates (Ferreira and Klein, 2011; Streltsov et al., 2011; Wilcox et al., 2011). While several studies have examined the neurotoxic potential of Aβ_42_ in several models, little is known about the effects of Aβ_40_ oligomers in human astrocytes.

Glutathione (GSH) is an important endogenous antioxidant found in millimolar concentrations in the brain. GSH levels have been shown to decrease with ageing and in several age-related degenerative diseases and AD in particular (Harris et al., 2015; Ilyas and Rehman, 2015; Romero-Haro and Alonso-Alvarez, 2015). Soluble oligomeric Aβ has been shown to induce oxidative stress and has been proposed to play a central role in the oxidative damage detected in AD brain (Xu et al., 2014). It has been shown that administration of γ-glutamylcysteine (GGC) increases cellular levels of GSH, circumventing the regulation of GSH biosynthesis by providing the limiting substrate (Nakamura et al., 2012; Quintana-Cabrera et al., 2012).

Whilst extracellular Aβ plaques, NFT, inflammation in the form or reactive astrocytes and microglia, and neuronal loss are all consistent pathological features of AD, a mechanistic link between these factors is yet to be clarified. Although most of the past research has focused on fibrillar Aβ, soluble oligomeric Aβ species are now considered to be of major pathological importance in AD. GGC is a dipeptide which exhibits potent antioxidant properties in several experimental models (Lai et al., 2008; Quintana-Cabrera et al., 2012). It serves as an essential cofactor for the antioxidant enzyme glutathione peroxidase (GPx) and is a precursor for GSH synthesis (Quintana-Cabrera et al., 2012). The metal-chelating properties of GGC have also been demonstrated previously (Salama et al., 2016).

In this study, we evaluated the protective role of up-regulation of GSH by GGC against biomarkers of apoptosis and cell death, oxidative stress, and neuroinflammation against oligomeric Aβ_40_ in primary human astrocyte cell cultures.

## Materials and Methods

### Cell Cultures

Human adult brain tissues were collected following surgical resection with both informed and written consent from patients who underwent surgery at the Centre for Mininally Invasive Neurosurgery, Prince of Wales Private Hospital, NSW, Australia. Healthy brain specimens were also acquired from patients during surgery for their tumor. A portion of the surgically resected brain tumor and healthy tissue sample was snap-frozen in liquid nitrogen immediately and stored at −80°C for prolonged storage for future use. Astrocytes were prepared from the mixed brain cell cultures using a protocol previously described by Guillemin et al. (2001).

Human astrocytes were pre-incubated for 15 min with 200 μM GGC. Afterward, cells were treated with oligomeric Aβ_40_ (10 nM, see Supplementary File for production and characterization), and biochemical assays were subsequently measured 24 h later. Experiments were performed in quadruplicates using cultures derived from three different human fetal brains.

### Extracellular LDH Activity as a Measurement for Cytotoxicity

The release of lactate dehydrogenase (LDH) into culture supernatant correlated with the amount of cell death and membrane damage, providing an accurate measure of cellular toxicity. LDH activity was assayed using a standard spectrophotometric technique described by Koh and Choi (1987).

### Caspase-3 Activity as a Measurement for Apoptosis

Caspase-3 activity is a well-established apoptotic biomarker. We quantified caspase-3 activity in brain cells using a commercially available kit (R&D systems) according to the manufacturer’s instructions. Briefly, an aliquot of cell homogenate was incubated with the labeled substrate DEVD-pNA (acetyl-Asp-Glu-Val-Asp p-nitroaniline), which is cleaved by the caspase-3 enzyme and releases the chromophore pNA. The levels of pNA were detected using the BMG Fluostar Optima multimode plate reader (NY, USA), at a wavelength of 405 nm.

### 7′-Dichlorofluorescin Diacetate (DCFDA) Assay for Production of Reactive Oxygen Species

Cells were incubated in 10 μM DCFDA (Sigma) in PBS at 37°C for 30 min as previously described (Chiu et al., 2013). Treatments were added in PBS. After addition of treatments, Fluorescence was measured at 0 min and 90 min with an excitation wavelength of 485 nm and an emission wavelength of 535 nm. The initial zero-point readings were subtracted from the 90 min readings as background.

### Quantification of 8-Hydroxy-2′-Deoxyguanosine (8-OH-dG) as a Marker for Oxidative DNA Damage

We used 8-Hydroxy-2′-Deoxyguanosine (8-OH-dG) as a biomarker for oxidative DNA on brain cells exposed to different treatments using the commercially available HT 8-OH-dG ELISA II kit (R&D Systems) as guided by the manufacturer’s instructions. Briefly, the 8-OH-dG monoclonal antibody binds competitively to the pre-coated 8-OH-dG as well as in the cell homogenate. Antibody bound to 8-OH-dG in the cell homogenate is washed away during washing and only antibody bound to the well was detected using a HRP-conjugate and colorimetric substrate and the BMG Fluostar Optima multimode plate reader (NY, USA).

### Measurement of Lipid Peroxidation

Lipid peroxidation level in the brain cell homogenate was quantified using the thiobarbituric acid reactive substances (TBARS) as previously described (Buege and Aust, 1978) and using the BMG Fluostar Optima multimode plate reader (NY, USA).

### Measurement of Protein Carbonyl Content

Protein carbonyls were used as a measure of protein oxidation, in brain cell homogenates using DNPH as previously described (Levine et al., 2000) and using the BMG Fluostar Optima multimode plate reader (NY, USA).

### Measurement of Superoxide Dismutase (SOD) and Glutathione Peroxidase (GPx) of the Antioxidant Enzymes Activity

Superoxide dismutase (SOD) and GPx activities were quantified using commercially available kits (Cayman chemicals) according to the manufacturer’s guidelines. The SOD assay is based on the formation of a formazan dye following exposure to superoxide anions (generated by hypoxanthine xanthine oxidase system) on a tetrazolium salt. The generated superoxide anions are dismutased by SOD leading to a reduction in the formation of formazan dye. The levels of formazan can be quantified at 450 nm was using the BMG Fluostar Optima multimode plate reader (NY, USA). GPx is based on the reduction of hydrogen peroxide by GPx present in the brain cell homogenate using GSH. Glutathione reductase (GR) and NADPH are then used to regenerate GSH. The assay quantified the levels of NADPH at 340 nm using the BMG Fluostar Optima multimode plate reader (NY, USA).

### Measurement of Total Antioxidant Capacity

Total antioxidant capacity (TAC) was assessed in brain cell homogenates using the commercially available kit (Cayman total antioxidant assay) in accordance to the manufacturer’s guide using the BMG Fluostar Optima multimode plate reader (NY, USA).

### Determination of Reduced GSH and Oxidized to Reduced GSH Ratio

The GSH assay method used in this study was adapted from Rahman et al. (2006) and all buffer formulations and assay reagent preparations were based on the original protocol. Briefly, neuronal homogenates extracts were diluted in Potassium phosphate-EDTA (KPE) buffer such that the sample values would fall within the standard curve range. Standards were prepared in matching dilutions of the lysis/extraction buffer in KPE buffer. Afterward, 20 μl of KPE buffer, serving as the blank, along with 20 μl of each of the standards, were dispensed into wells of a single row of a 96-well microplate. The assay’s designated standard range was between 0.1 through to 46.0 nM. The plate was loaded into BMG’s Fluostar Optima multimode plate reader (NY, USA), controlled by native software (version 2.10 R2). Both pumps were employed to dispense assay reagents, with Pump 1 being used to deliver a solution of equal parts GR and 5,5′-dithiobis-(2-nitrobenzoic acid; DTNB) in KPE. Pump 2 was used to deliver β-NADPH in KPE. One-hundred and twenty microliter of the combined GR and DTNB reagent and 60 μl of β-NADPH reagent was dispensed per well, into wells containing samples/standards. The rate of chromogenic TNB (5-thio-2-nitrobenzoic acid) formation from the non-chromogenic substrate DTNB [5,5′-dithio-bis (2-nitrobenzoic acid)] is directly proportional to the amount of GSH present and can be measured at 412 nm. Oxidized to reduced GSH ratio was determined using GSSG/GSH detection kit (Enzo Diagnostics, New York, NY, USA) as described in the manufacturer’s guidelines.

### Quantification of a Panel of Inflammatory Cytokines

The levels of the anti-inflammatory cytokine IL-10 and the pro-inflammatory cytokines TNF-α, IL-6, and IL-1β in brain cell homogenates were quantified using specific ELISA kits (Ray Biotech, Peachtree Corners, GA, USA) according to the manufacturer’s instructions. These assays use biotinylated antibodies and streptavidin–HRP conjugate and a TMB (3,3′,5,5′-tetramethylbenzidine)-based detection system.

### Measurement of Matric Metalloproteinase Activity (MMP-2 and MMP-9)

Matric metalloproteinase (MMP)-2 and MMP-9 activities were quantified using commercially available kits (AnaSpec) according to the manufacturer’s guidelines. The MMP-2 and MMP-9 activity assays use a 5-FAM (fluorophore) and QXL520™ (quencher) labeled FRET peptide substrate to quantify MMP-2 and MMP-9 activity in the sample. Cleavage of the FRET peptide by MMP-2 and MMP-9 alters the fluorescence of 5-FAM which can be quantified at excitation/emission of 490 nm/520 nm using the BMG Fluostar Optima multimode plate reader (NY, USA).

### Bradford Protein Assay for the Quantification of Total Protein

LDH, caspase 3, SOD and GPx activities, the levels of DCFDA, 8-OH-dG, TBARS, DNPH, GSSG/GSH and several cytokines, and TAC were adjusted for variations in cell number using the Bradford protein assay described by Bradford (1976).

### Data Analysis

The results obtained are presented as the means ± the standard error of measurement (SEM). One-way analysis of variance (ANOVA) and *post hoc* Tukey’s multiple comparison tests were used to determine statistical significance between treatment groups. Differences between treatment groups were considered significant if *p* was less than 0.05 (*p* < 0.05).

## Results

### Characterization of Recombinant Oligomeric Aβ_40_

Recombinant preparations of oligomeric Aβ_40_ were prepared and characterized using atomic force microscopy (AFM). Supplementary Figure S1A shows oligomeric Aβ_40_ as evidenced by a homogeneous population of spherical particles averaging 3–5 nm in size. No mature amyloid fibril structures were observed in these preparations.

We further characterized our oligomeric Aβ_40_ preparation using western blotting (Supplementary Figure S1B) using the monoclonal antibody 6E10 recognizing residues 1–17 of Aβ. Recombinant oligomeric Aβ_40_ preparations contained large amounts of low molecular weight species corresponding to monomer, dimer, trimer and tetramer, with a smear of Aβ species between 16 kDa and 50 kDa. Similar trends in migration patterns for oligomeric Aβ_40_ have been observed in other laboratories using the same Aβ aggregation method (Dahlgren et al., 2002). However, other laboratories have also reported detection of a larger molecular weight oligomeric Aβ species in their recombinant preparations that range from 40–98 kDa (Garzon and Fahnestock, 2007). Differences in these results may be due to variations in western blotting techniques used from laboratory to laboratory, for example, the specific composition of gels and the running buffer/s used.

### Neurotoxic Effect of Oligomeric Aβ_40_ in Isolated Adult Astrocytes Can be Ameliorated Using GGC

**Figure 1:**
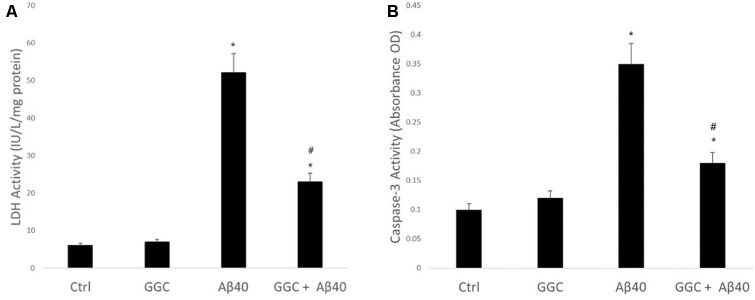
Neurotoxic effect of oligomeric Aβ_40_ in isolated adult astrocytes can be ameliorated using GGC. **(A)** Aβ_40_ significantly decreased astrocyte cell viability. Treatment with GGC (200 mM) provided significant protection from exposure to oligomeric Aβ_40_. **(B)** Treatment of astrocytes with GGC significantly reduced caspase 3 activity. Significance *p < 0.05 compared to control non-treated cells. ^#^*p* < 0.05 compared to control cells treated with oligomeric Aβ_40_.

Figure 1A shows the effect on neuronal viability, assessed by release of LDH into the media of human primary astrocytes following treatment with 10 nM oligomeric Aβ_40_ for 24 h. Oligomeric Aβ_40_ significantly decreased astrocyte cell viability. Treatment with GGC (200 μM) provided significant protection from exposure to oligomeric Aβ_40_. Treatment of astrocytes with GGC significantly reduced caspase 3 activity (Figure 1B), supporting the protective effects of GGC against oligomeric Aβ_40_-mediated apoptosis.

### Oligomeric Aβ_40_ Induces Free Radical Damage to DNA, Lipids, and Protein Which Could be Attenuated Using GGC

**Figure 2:**
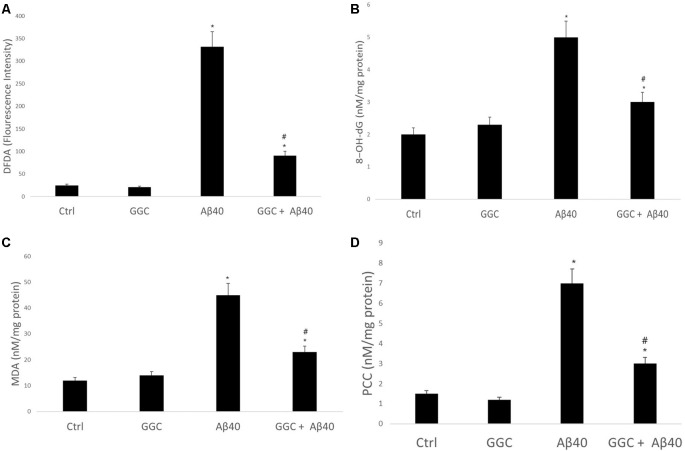
GGC attenuates oligomeric Aβ_40_-mediated oxidative stress production and oxidative damage to DNA, lipids, and protein in isolated adult astrocytes. **(A)** Oligomeric Aβ_40_ increased reactive oxygen species production using the DCFDA Assay, and which was reduced by treatment with GGC. Co-treatment with GGC also ameliorated **(B)** oxidative DNA damage, **(C)** lipid peroxidation, and **(D)** protein carbonyl formation. Significance *p < 0.05 compared to control non-treated cells. ^#^*p* << 0.05 compared to control cells treated with oligomeric Aβ_40_.

To confirm that oligomeric Aβ_40_ is cytotoxic to astrocytes *via* induction of oxidative stress, we monitored the effect of oligomeric Aβ_40_ on free radical generation. Figure 2A demonstrates that incubation with oligomeric Aβ_40_ can increase reactive oxygen species production using the DCFDA Assay, and which was reduced by treatment with GGC. Co-treatment with GGC ameliorated oxidative DNA damage (Figure 2B), lipid peroxidation (Figure 2C), and protein carbonyl formation (Figure 2D).

### Oligomeric Aβ_40_ Depletes Endogenous GSH Levels Which Can be Attenuated Using the GGC

**Figure 3:**
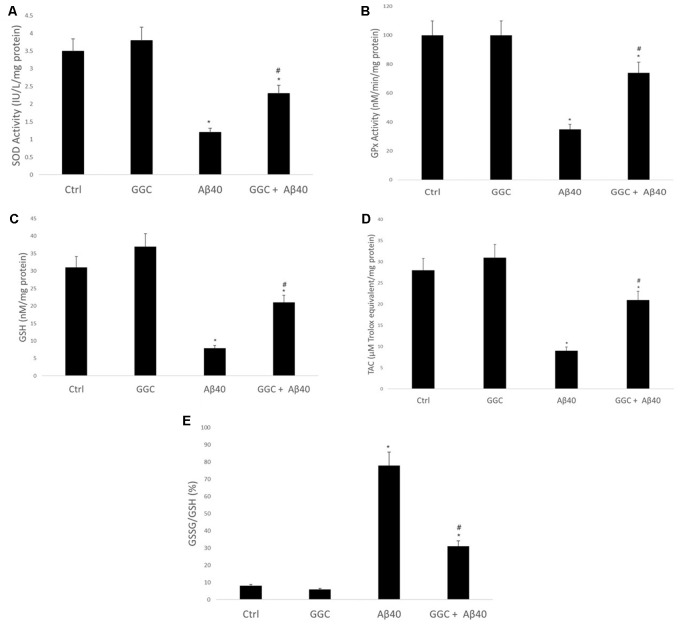
GGC increases **(A)** SOD and **(B)** GPx activities and **(C)** total antioxidant capacity, **(D)** attenuates oligomeric Aβ_40_-mediated GSH depletion, and **(E)** decreases the GSSG/GSH ratio in isolated adult astrocytes. Significance *p < 0.05 compared to control non-treated cells. ^#^*p* < 0.05 compared to control cells treated with oligomeric Aβ_40_.

Oligomeric Aβ_40_ decreased the activity of the antioxidant enzymes SOD (Figure 3A) and GPx (Figure 3B) compared to non-treated cells. Similarly, it reduced the levels of the GSH (Figure 3C) and the TAC (Figure 3D) in brain cell homogenates and increased the ratio GSSG/GSH (Figure 3E). Co-administration with GGC significantly increased the activity of SOD and GPx. Similarly, GGC increased the levels of GSH and TAC and significantly reduced the GSSG/GSH ratio. Therefore, GGC may attenuate oligomeric Aβ_40_-mediated disruption of endogenous antioxidant enzymes and replenish GSH levels in human brain cells.

### Oligomeric Aβ_40_ Induces Inflammation Which Is Modulated by GGC

**Figure 4:**
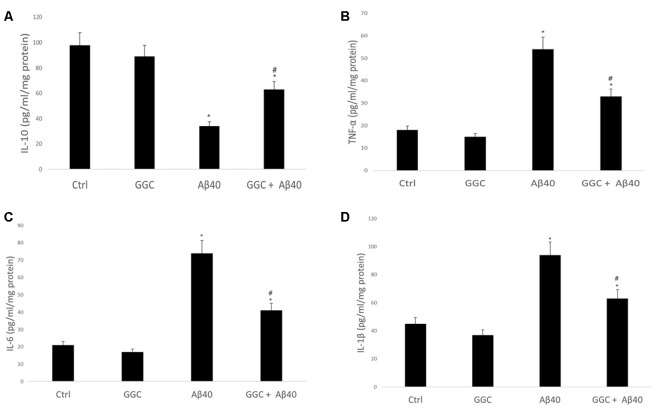
GGC increases **(A)** the levels of the anti-inflammatory cytokine IL-10 and decreases the levels of pro-inflammatory cytokines, **(B)** TNF-α, **(C)** IL-6 and **(D)** IL-β in isolated adult astrocytes. Significance *p < 0.05 compared to control non-treated cells. ^#^*p* < 0.05 compared to control cells treated with oligomeric Aβ_40_.

Treating human brain cells with oligomeric Aβ_40_ significantly decreased the level of the anti-inflammatory cytokine IL-10 (Figure 4A) and increased the levels of the pro-inflammatory cytokines, TNF-α, IL-6 and IL-1β compared to non-treated cells (Figures 4B–D). GGC effectively modulated the observed oligomeric Aβ_40_-induced changes in the levels of inflammatory cytokines *in vitro*. We observed a significant upregulation in IL-10 levels and significant downregulation of TNF-α, IL-6, and IL-1β in GGC-treated cells, suggestive of the beneficial effects of GGC against oligomeric Aβ_40_-induced neuroinflammatory response.

### GGC Supplementation Reduces Metalloproteinase Activity Induced by Oligomeric Aβ_40_

**Figure 5:**
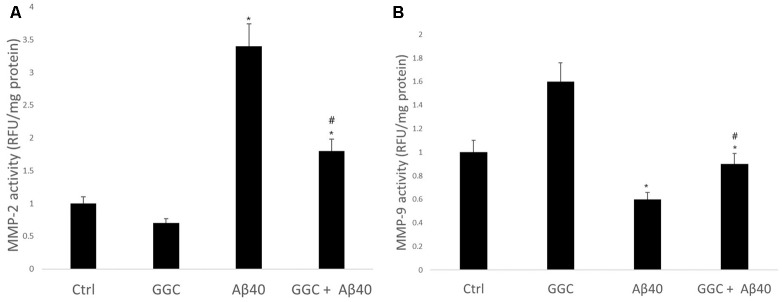
GGC reduces **(A)** MMP-2 and **(B)** MMP-9 activities and attenuates oligomeric Aβ_40_-induction of metalloproteinase activity in isolated adult astrocytes. Significance *p < 0.05 compared to control non-treated cells. ^#^*p* << 0.05 compared to control cells treated with oligomeric Aβ_40_.

Treating human brain cells with oligomeric Aβ_40_ significantly increased the activity of MMP-2 (Figure 5A) and decreased the activity of MMP-9 compared to non-treated cells (Figure 5B). GGC attenuated the effect of oligomeric Aβ_40_-induced changes in MMP-2 and MMP-9 activities *in vitro*. We observed a significant decrease in MMP-2 activity and a significant increase in MMP-9 activity in GGC-treated cells, suggestive of the differential effect of GGC and increased GSH on MMPs.

## Discussion

GSH is the most potent antioxidant in the human body. It has multiple antioxidant actions which include direct conjugation with free radicals, enzyme-mediated neutralization of free radicals, and the regeneration of other eminent antioxidants such as Vitamin C. Owing to its potent biological availability, replenishing GSH levels following GSH depletion is an important therapeutic target. In this study, we attempted to evaluate whether the GSH precursor, GGC, could increase GSH levels in primary astrocytes and protect primary astrocytes against biomarkers of apoptosis and cell death, oxidative stress, and neuroinflammation when exposed to pathophysiological concentration of oligomeric Aβ_40_.

Aβ is a peptide that is formed following the proteolytic cleavage of the amyloid precursor protein (APP; Murphy and LeVine, 2010). It forms the principal component of extracellular amyloid plaques which are one of the main pathological hallmarks of AD. While several Aβ species with variable lengths (38–43 amino acids) have been identified, Aβ_40_ is thought to be most abundant (80%–90%) followed by Aβ_42_ (5%–10%; Selkoe, 2001). These peptides have been localized in amyloid plaques and can form oligomers and protofibrils, although Aβ_42_ is more hydrophobic and fibrillogenic (Selkoe, 2001). Post-mortem analysis of human brains from AD patients showed that that Aβ_40_ discriminated between AD patients and high pathology controls more readily than Aβ_42_ (Gao et al., 2010). Aβ_40_ oligomers have been shown to induce neurotoxicity and disrupt brain lipid bilayers while other amyloid species do not (Bode et al., 2019). Aβ_40_ is induced by an increase in intracellular Ca^2+^, and this has led to the hypothesis that the accumulation of intracellular Aβ may occur as an early event in the pathogenesis of AD (Itkin et al., 2011). Therefore, aggregation of intracellular Aβ may be an adaptive response to increases in [Ca^2+^]_i_ and may enhance Ca^2+^-mediated excitotoxicity, which may induce progressive memory loss and cognitive deficits and enhance neuronal cell apoptosis. This can explain why ageing is a major risk factor for AD, since when calcium imbalance is more pronounced with advanced age.

Our data show that addition of Aβ_40_ oligomers increased cytotoxicity in astrocytes after a 24-h incubation. On the contrary, treatment of astrocytes with GGC attenuated cell viability and decreased oligomeric Aβ_40_-mediated oxidative stress and release of pro-inflammatory cytokines *in vitro* after 24 h. Our results suggest that GGC can enter astrocytes and increase GSH levels and reduce the GSSG/GSH ratio, which in turn, attenuates intracellular oxidative stress and protect astrocytes from oligomeric Aβ_40_-mediated oxidative stress and inflammatory response.

MMPs are important proteolytic enzymes necessary for the maintenance of the integrity of the extracellular matrix. Under normal physiological conditions, MMPs are secreted as inactive zymogens. However, under pathological conditions, activated MMPs are associated with increased degradation of ECM components and are involved in ECM re-modeling (Dollery et al., 1995). MMP2 has been reported to be a key player in the disruption of tight junction proteins and enhance blood brain barrier (BBB) permeability (Yang et al., 2007). Consistent with these findings, our data shows that Aβ_40_ oligomers can increase MMP-2 activity in stimulated astrocytes and correlated with increased oxidative stress, inflammation and reduced cell viability. MMP-9 has been shown to degrade fibrillar deposits and is co-localized with vascular amyloid deposits and neurofibrillary tangles (Alvarez-Sabín et al., 2004). However, since only the latent form of MMP-9 has been shown to accumulate in the AD brain, it has been hypothesized that impaired MMP-9 activity may lead to accumulation of abnormal Aβ peptides in amyloid plaques (Lim et al., 1996). With regards to this hypothesis, our data shows that Aβ_40_ oligomers reduce MMP-9 activity in human astrocytes and MMP-9 activity is increased following treatment with GGC. Our results are in line with previous findings showing the direct inhibitory effect of GSH on MMP-2 gelatinolytic capacity, and GSH-mediated increases in MMP-9 gelatinolytic activity *in vitro* (Bogani et al., 2007). The same study reported upregulation of MMP-9 transcription and activity following treatment with GSH (Bogani et al., 2007), and we observed a similar finding in GGC treated cells alone. Therefore, the differential effect of GGC on MMPs is likely to reduce ECM breakdown and promote degradation of Aβ peptide and may be dependent on GSH status.

We additionally found that GGC can increase intracellular GSH levels and lower the GSSG/GSH ratio in astrocytes. Alterations in the levels of either reduced (GSH) or oxidized (GSSG) GSH may affect the function of insulin-degrading enzyme (IDE) which is involved in mediating insulin degradation (Kulas et al., 2019). IDE is also a major regulator of Aβ peptides and reduced IDE activity can reduce Aβ degradation by more than 50% (Farris et al., 2003). One study demonstrated the inhibitory effects of GSSG on IDE activity, potentially due to antagonism of substrate binding. However, GSH enhanced IDE activity non-enzymatically by reducing exposed disulfide bonds and stimulating insulin breakdown (Cordes et al., 2011). Therefore, the intracellular GSH/GSSG ratio may affect the processing and degradation of both insulin and Aβ. Further studies are necessary to confirm whether GGC can increase IDE activity due to increased GSH levels, and may represent an additional mechanism to explain the potential neuroprotective effects of GGC in AD.

**Figure 6:**
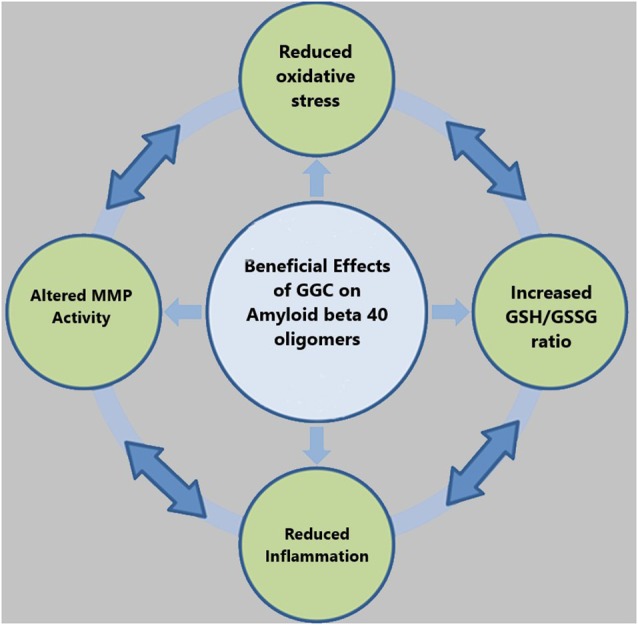
GGC can protect against oligomeric Aβ_40_ toxicity by reducing oxidative stress and increasing GSH levels.

GGC is a dipeptide containing cysteine and glutamic acid. Apart from the metal chelating activity of cysteine residue that has been demonstrated in N-acetylcysteine (NAC; Sevgiler et al., 2011), glutamic acid has been reported to chelate redox-active metals. Therefore, the presence of both cysteine and glutamic acid residues in GGC may be likely responsible for its potent antioxidant and anti-inflammatory effects in brain cells against insult oligomeric Aβ_40_. In general, dipeptides have been considered unlikely to be effective as oral drug candidates, since they are prone to hydrolysis by digestive or serum proteases. However, GGC consists of a gamma-glutamyl bond which makes it resistant to hydrolysis by endoproteases (Anderson and Meister, 1983). As well, the importance of GGC as a cofactor for GPx to increase GSH levels is also another mechanism for the protective effects of GGC in human brain cells. The observed immunomodulatory effects of GGC on oligomeric Aβ_40_-induced inflammation may, thus, be also due to chelation of redox-active metals and additional antioxidant mechanisms (Figure 6).

The effects of GGC in human astrocytes is relevant for neuroprotection in AD and other neurodegenerative diseases since astrocytes represent the supporting cells in the brain and protect neurons against oxidative stress and enhance neuronal survival during cytotoxic conditions. It has been demonstrated that GSH depleted astrocytes lose their neuroprotective roles, leading to neuronal toxicity and a consequent reduction in cell viability (Pizzurro et al., 2014a,b). Depletion of GSH in astrocytes is also deleterious to astrocytes themselves (Im et al., 2006; Oz et al., 2006). Astrocytes are responsible for the clearance of debris in extracellular space and are required to maintain blood-brain barrier stability (He et al., 2008). The loss of astrocyte function can compromise the function and structure of neurons, culminating in neurodegeneration, and overall brain dysfunction (Schilling et al., 2006). Thus, maintenance of optimal GSH levels is necessary for neuroprotection against oxidative stress and neuroinflammation in astrocytes by maintaining a healthy environment in the CNS.

Strategies aimed at elevating GSH levels have been met with mixed success. The limiting factors include: (1) the inability of GSH to cross the cell membrane *via* direct absorption; (2) feedback inhibitory effects of GSH on glutamate cysteine ligase (GCL) activity (the first biosynthetic enzyme), which is regulated by the availability of cysteine (Jones, 2011); and (3) reduced activity of GCL with advanced age (Liu and Dickinson, 2003). Moreover, several cysteine-based antioxidant trials on AD patients have been unsuccessful (Berk et al., 2013). The therapeutic potential for GGC to increase GSH is related to the second GSH biosynthetic enzyme, glutathione synthetase (GS) which is not regulated by non-allosteric feedback inhibition by GSH. GS catalyzes the addition of glycine to GGC to produce GSH (Zarka and Bridge, 2017). This provides support for the benefits of GGC as an immediate precursor to GSH.

Direct delivery of GCC to the brain has been previously reported to protect against cellular depletion of GSH in the brain using the Buthionine sulfoximine (BSO) model of depletion and increase GSH levels in the pre-perturbed model (Pileblad and Magnusson, 1992; Dringen et al., 1997). It has been reported that the cytosolic concentrations of GGC are close to 7 μM, and passive uptake of intact GGC can be directed into cells. Reduced GSH levels due to impaired GCL activity is unlikely to be replenished by other GSH precursors such as NAC or other cysteine prodrugs. Supplementation with GGC is likely to increase GS activity and attenuate GSH depletion.

Previous studies such as those by Dringen et al. (1997) relied on limiting or sub-physiological conditions to examine the effects of GGC on increases in GS activity in astrocyte-rich primary cultures. However, these do not accurately represent healthy pre-pathological conditions and are unlikely to add support to the ability of GGC to bypass feedback inhibition of GCL. Le et al. (2011) showed that 2-h GGC treatment at 100 or 500 μM, following 4-h exposure to H_2_O_2_, could not significantly replenish GSH levels or increase GSH levels above homeostatic levels in murine neurons and astrocyte cell cultures. However, in that study, the neuronal and glial cells were co-treated with cytotoxic concentrations of H_2_O_2_, and therefore it is unlikely that GGC could produce beneficial effects on GSH in this condition. As well, that study could be improved by adding an additional control condition which was exposed to GGC in the absence of H_2_O_2_ insult.

Recently, our group showed that orally dosed GGC (2 and 4 g) is bioavailable and can increase intracellular GSH levels above homeostasis in lymphocytes of healthy, non-fasting human subjects with no adverse effects (Zarka and Bridge, 2017). This suggests that GGC may increase GSH levels in the clinic with potential as adjunct therapy for the treatment of disorders associated with acute and/or chronic GSH depletion. Previous preclinical studies have demonstrated little or no toxicity following administration of GGC (Joshi et al., 2007; Espinosa-Diez et al., 2015; Zhang et al., 2015; Ding et al., 2016; Henderson et al., 2016; Salama et al., 2016). The present study set out for the first time to evaluate GGC as a GSH-elevating strategy in primary human astrocytes, starting from homeostatic levels, with promising data.

In conclusion, this study has shown that GGC can decrease apoptosis, oxidative stress and inflammation in human astrocytes and protect astrocytes from oligomeric Aβ_40_-mediated cytotoxicity cellular GSH depletion. The beneficial effects of GGC on human brain cells may prevent against neurodegeneration by increasing the availability of GSH precursors, which would contribute to the neuroprotective effects of GGC in AD.

## Data Availability

All datasets generated for this study are included in the manuscript and/or the Supplementary Files.

## Ethics Statement

The approval for this study was obtained from the Human Research Ethics Committee of the University of New South Wales (human brain tissue reference number HC12563).

## Author Contributions

NB, DC, and PS formulated the present hypothesis. NB was responsible for writing the report. NB, MZ, B-EJ, JW, TJ, and WB performed the experiments.

## Conflict of Interest Statement

The authors declare that the research was conducted in the absence of any commercial or financial relationships that could be construed as a potential conflict of interest.
