# Supplementary material for: The Precursor to Glutathione (GSH), γ-Glutamylcysteine (GGC), Can Ameliorate Oxidative Damage and Neuroinflammation Induced by Aβ40 Oligomers in Human Astrocytes
Source: Front Aging Neurosci. 2019 Aug 8;11:177. doi: 10.3389/fnagi.2019.00177 (PMC6694290; doi:10.3389/fnagi.2019.00177)
Supplement: Supplementary file 1 [file Data_Sheet_1.docx]

**Supplementary File**

**Preparation of recombinant Aβ_40_ Peptide**

Aβ_40_ peptide was purchased from Recombinant Peptide Technologies (Athens, GA, USA). The peptide was stored in sealed glass vials at -80^o^C in a lyophilised form. Recombinant Aβ_40_ oligomers were prepared as previously described (Dahlgren, Manelli et al. 2002).

**Solubilisation of Aβ recombinant peptide**

The lyophilised peptide was dissolved in 1mM 1,1,1,3,3,3-hexafluoro-2-propanol (HFIP; Sigma, Castle Hill, Australia) and separated into 50μl aliquots in sterile microcentrifuge tubes. The aliquots were left for three hours in the fume hood, to allow the HFIP to evaporate. The peptide film was dried under vacuum using a Speed Vac (ThermoSavant, Patterson, CA, USA), and stored at -20^o^ C until required.

**Production of recombinant Aβ_40_ Oligomers**

The peptide film prepared as described above was resuspended to 5mM in anhydrous dimethyl sulfoxide (DMSO; Sigma, Castle Hill, Australia). Ice-cold Dulbecco's Modified Eagle Medium/Ham F-12 without phenol red (Sigma, Castle Hill, Australia) was added to a final concentration of 100μM. Afterwards, the sample was vortexed for 30 seconds and incubated at 4^o^C for 6 weeks.

**Characterisation of recombinant oligomeric Aβ_40_ by atomic force microscopy**

Aβ_40_ oligomers were diluted 1:10 using deionised filtered water. 25μL of the Aβ preparations was added onto freshly cleaved mica and incubated at room temperature for 10 minutes. After careful washing with 50μl of deionised filtered water to remove any unbound peptide, the sample was dried for AFM analysis using a Nanoscope III Scanning Probe Microscope (Digital Instruments Extended Dimension 3000, Santa Barbara, CA, USA).

**Characterisation of recombinant oligomeric Aβ_40_ preparations by Western blotting**

Aβ_40_ oligomers (600μM) were diluted with 4x Lamelli loading buffer (Hercules, CA), boiled for 5 min, electrophoresed on 8–12% (v/v) polyacrylamide SDS-PAGE gels (BioRad, Hercules, CA, USA), and electrotransferred onto PVDF membranes (BioRad, Hercules, CA, USA), and the membrane blocked with 5% non-fat milk dissolved in TBS for 1 h and incubated with 6E10 (1:1000), a mouse monoclonal Aβ antibody to residues 1-17 (Sigma, Castle Hill, Australia) overnight at 4˚C. After incubation with the primary antibody, membranes were washed in TBS-Tween-20 and incubated with horseradish peroxidase (HRP) conjugated IgG anti-mouse secondary antibody (1:5000, Sigma, Australia) for 1 h at ambient temperature. After further washing in TBS-Tween-20, the membranes were incubated with an ECL plus reagent (RPN2132, Amersham) and protein bands were visualised on X-ray films. The molecular mass was estimated using SeeBlue®Plus 2 Standard molecular weight markers (Bio-Rad Laboratories).

**Figure 1. Recombinant preparations of oligomeric Aβ_40_. (A) Characterisation of recombinant oligomeric Aβ_40_ by atomic force microscopy**. **(B) Characterisation of recombinant oligomeric Aβ_40_ preparations by Western Immunoblotting Western**

**A.**

**
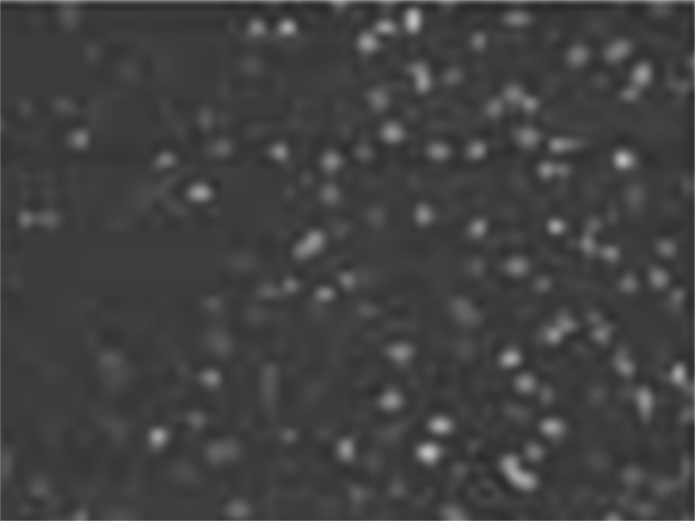
**

**1 µm**

**B.**


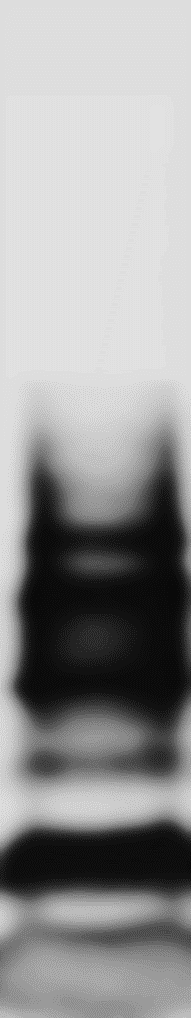

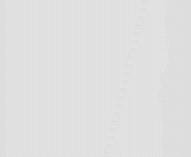


50 kDa

16 kDa

12 kDa

8 kDa

4 kDa

**References**

Dahlgren, K. N., A. M. Manelli, W. B. Stine, Jr., L. K. Baker, G. A. Krafft and M. J. LaDu (2002). "Oligomeric and fibrillar species of amyloid-beta peptides differentially affect neuronal viability." J Biol Chem **277**(35): 32046-32053.
